# In Vitro Screening and Transfection Concentration Optimization of Cynomolgus Monkey I*κ*B*α*-siRNA

**DOI:** 10.1155/2020/1848540

**Published:** 2020-04-15

**Authors:** Zhaoxing Ou, Rui Zeng, Yifan Lin, Si Zhang, Mohammad Alzogool, Peng Zeng, Yuqing Lan

**Affiliations:** Department of Ophthalmology, Guangdong Provincial Key Laboratory of Malignant Tumor Epigenetics and Gene Regulation, Sun Yat-sen Memorial Hospital, Sun Yat-sen University, Guangzhou 510120, China

## Abstract

**Purpose:**

To seek for a small interfering RNA (siRNA) sequence targeting a cynomolgus monkey inhibitor of nuclear factor kappa B *α* (I*κ*B*α*) that can specifically and effectively suppress I*κ*B*α* gene expression of cynomolgus monkey ciliary muscle (CM) cells and trabecular meshwork (TM) cells in vitro and screen for optimal siRNA transfection concentration.

**Methods:**

Three I*κ*B*α*-specific double-stranded siRNAs were designed and synthesized. They were transfected into primarily cultured cynomolgus monkey CM cells and TM cells. The mRNA and protein levels of I*κ*B*α* were examined by using real-time quantitative polymerase chain reaction (real-time PCR) and western blot to screen a pair of candidate valid sequences with the highest inhibitory rate. Both cells were transfected with Cy5-labeled nonspecific control-siRNA (NC-siRNA) of four different concentrations (10, 20, 50, and 100 nmol/L(nM)), and flow cytometry was used to assess transfection efficiency. Then, cells were transfected with the candidate valid I*κ*B*α* -siRNA of the same four concentrations, and the cytotoxicity was detected by using Cell Counting Kit-8 (CCK8), and the inhibitory efficiency of I*κ*B*α* was identified via real-time PCR to find out optimal siRNA transfection concentration.

**Results:**

The suppression effect of the siRNA targeting the GCACTTAGCCTCTATCCAT of I*κ*B*α* gene was most obvious by in vitro screening. The inhibitory rate of I*κ*B*α* was 82% for CM cells and 82% for TM cells on the mRNA level and 98% for CM cells and 93% for TM cells on the protein level, respectively. The results of flow cytometry showed that the transfection efficiency was the highest at 100 nM, which was 89.0% for CM cells and 48.2% for TM cells, respectively. The results of CCK8 showed that there was no statistically significant difference in cell viability after transfection of different concentrations of I*κ*B*α*-siRNA. The results of real-time PCR indicated that there was no statistical difference in the inhibitory efficiency of I*κ*B*α* after transfection of different concentrations of I*κ*B*α*-siRNA.

**Conclusion:**

It proves that the siRNA targeting the GCACTTAGCCTCTATCCAT of I*κ*B*α* gene is the valid sequence to suppress cynomolgus monkey I*κ*B*α* expression of CM cells and TM cells by RNAi. 10 nM is the optimal transfection concentration.

## 1. Introduction

Glaucoma is the second irreversible blinding eye disease in the world [1, 2]. The vast majority of glaucoma is caused by an increase in intraocular pressure due to increased resistance to aqueous outflow [1]. Studies have shown that matrix metalloproteinases (MMPs) can improve the aqueous humor outflow of the trabecular meshwork pathway and the uveoscleral pathway [3–7]. However, its upstream regulation mechanism is still a matter of debate. Nuclear factor kappa B (NF-*κ*B), a nuclear protein factor present in a variety of cells, regulates transcription and expression of genes, and participates in immune, inflammatory, stress, and other cellular processes, and meanwhile involves in pathophysiological processes such as differentiation, proliferation, and apoptosis [8]. The NF-*κ*B system consists of NF-*κ*B and its inhibitor (inhibitor of NF-*κ*B, I*κ*B). I*κ*B*α* is the earliest and most well-known member of the I*κ*B family [9]. We have demonstrated that I*κ*B*α*-siRNA could be transfected into normal human CM cells in vitro by RNA interference (RNAi), which downregulated I*κ*B*α* expression and promoted transcriptional activity of NF-*κ*B, consequently increasing expression of MMP-2 and decreasing expression of tissue inhibitors of metalloproteinase-2 (TIMP-2) [10]. We further transfected I*κ*B*α*-siRNA into rat ciliary muscles by anterior chamber injection and found that the expression of I*κ*B*α* was reduced, NF-*κ*B got activated, and expression and activity of MMP-2 got improved. In addition, intraocular pressure was lowered in rats. Besides, after the anterior chamber was injected with the Cy3-labeled I*κ*B*α*-siRNA for 24 hours, most of the red fluorescence accumulation was observed in the rat ciliary muscles, and a small amount was found in the trabecular meshwork, suggesting that I*κ*B*α*-siRNA might have an effect on the aqueous humor outflow dual pathways. In order to further study the cynomolgus monkey in vivo, we designed and synthesized 3 pairs of small siRNA targeting cynomolgus monkey I*κ*B*α* and transfected them into the cynomolgus monkey CM cells and TM cells. Real-time PCR and western blot were used to detect the expression of I*κ*B*α* mRNA and protein to screen the siRNA sequences which could effectively inhibit the expression of I*κ*B*α*. The transfection efficiency of different concentrations of siRNA was measured by flow cytometry. The cytotoxicity was detected by cck8, and real-time PCR was used to test the inhibitory rate of I*κ*B*α* after transfection of different concentrations of siRNA. These three methods were used to search for the optimal transfection concentration. This study would lay the foundation for further exploring the role of the NF-*κ*B/I*κ*B signaling pathway in the aqueous humor outflow dual pathways in glaucomatous cynomolgus monkeys.

## 2. Materials and Methods

All experiments on animals were conducted in accordance with the Association for Research in Vision and Ophthalmology Statement for the Care and Use of Animals in Ophthalmic and Vision Research. The research protocol was approved by the Animal Care Committee of the Sun Yat-sen Memorial Hospital at Sun Yat-sen University in China.

### 2.1. Primary CM Cells and TM Cells

Four healthy male cynomolgus monkeys, aged 3 to 4 years and weighing about 3.5 kg, were purchased from Guangdong Chunsheng Biotechnology Development Co., Ltd. Monkeys were killed by intravenous overdose of sodium pentobarbital. Eyes were then nucleated and stored at 4°C. CM cells and TM cells were prepared as previously described [11]. The medium was composed of Dulbecco's modified Eagle's medium and Ham's F12 nutrient mixture (DMEM/F12) (Gibco, USA), supplemented with 20% fetal bovine serum (FBS) (Gibco, USA), 1% penicillin-streptomycin (Hyclone, USA), and 1 ng/mL recombinant human basic fibroblast growth factor (bFGF) (Gibco, USA). The cultures were incubated in a 37°C humidified incubator with an atmosphere of 20% O_2_ and 5% CO_2_. The medium was changed every 3 or 4 days. After the primarily cultured cells reached confluence, subsequent passages were performed. The confluent fourth to sixth passaged cells were used in the subsequent study.

### 2.2. Identification of Cultured Cells

To identify CM cells, anti-*α* smooth muscle actin (*α*-SMA) antibody (Bioss, China) was used for the immunofluorescence study. Meanwhile, anti-laminin (LN) antibodies (Bioss, China), anti-fibronectin (FN) antibodies (Bioss, China), and anti-neuron-specific enolase (NSE) antibodies (Bioss, China) were used for the identification of TM cells. Cultured cells were inoculated on the cell slides for 80% to 90% confluence. They were fixed in a 4% paraformaldehyde solution (Bestbio, China) for 15 minutes and then were permeabilized in 0.5% Triton X-100 (Solarbio, China) for 20 minutes. Then, they were blocked with normal goat serum (Bioss, China) for 10 minutes and incubated at 37°C in the presence of anti-*α*-SMA/LN/FN/NSE antibody diluted at 1 : 50 overnight. The specimens were then incubated for 30 minutes at 37°C in the presence of Cy3-conjugated goat anti-rabbit IgG antibody (Jackson, USA) diluted at 1 : 100. Then, they were incubated with DAPI (Bestbio, China) diluted at 1 : 50 in the dark for 5 minutes. The antifluorescence attenuating capsules (Solarbio, China) were mounted, and the images were taken under a fluorescence microscope (Carl Zeiss, Germany).

### 2.3. Design and Synthesis of I*κ*B*α*-siRNA

We queried the mRNA sequence of cynomolgus I*κ*B*α* gene (GenBank NM_001284932.1) in the NCBI gene pool of the National Center for Biotechnology Information. Three pairs of siRNA against I*κ*B*α* gene (Table 1), a pair of nonspecific control-siRNA (NC-siRNA), and a pair of Cy5-labeled NC-siRNA, all 19 bp in length, were designed and chemically synthesized by Guangzhou Ruibo Biotech Co., Ltd, China.

### 2.4. Screening of I*κ*B*α*-siRNA Sequences

#### 2.4.1. Preparation and Transfection of siRNA

Both cells were divided into 4 groups: negative control group transfected with NC-siRNA and experimental groups (Nos. 1, 2, and 3) transfected with three pairs of I*κ*B*α*-siRNA, respectively. The cells were digested by conventional methods and inoculated in a 6-well culture plate at about 5 × 10^5^ cells per well. When the cell fusion degree got 60–80%, the medium was replaced by fresh DMEM/F12 medium containing 20% FBS (without antibiotics). NC-siRNA and three pairs of I*κ*B*α*-siRNA were prepared at the final concentration of 10 nM with Opti-MEM (Invitrogen, USA) according to operating instructions of Lipofectamine® RNAiMAX Reagent (Invitrogen, USA). NC/I*κ*B*α*-siRNA and the transfection reagent were mixed for 5 minutes and then were transfected into cells.

#### 2.4.2. Real-Time PCR Analysis

Forty-eight hours after transfection, the total RNA was extracted from each group by using RNA Quick Purification kit (ESscience, China). The concentration and purity of RNA samples were determined by UV spectrophotometry. The total RNA of each group was reversely transcribed to generate cDNA with PrimeScript™ RT Master Mix (Perfect Real Time) (Takara, Japan). Real-time PCR was performed on I*κ*B*α* and ACTB (internal control) with Green Premix Ex Taq II (Tli RNaseH Plus) (Takara, Japan). The PCR reaction conditions were as follows: predenaturation at 95°C for 30 seconds, denaturation at 95°C for 5 seconds, and annealing at 60°C for 30 seconds, for a total of 40 cycles. Cynomolgus monkey I*κ*B*α* and ACTB primers were designed and synthesized by Shanghai Biotech Co., Ltd, China, and homology analysis was performed on BLAST. Primer sequence of I-*κ*B*α*: F:5′-CTGGTGTCGCTCCTGTTGAAGTG-3′, 23 bp; R:5′-TGTCATAGCTCTCCTCATCCTCACTC-3′, 26 bp; internal reference ACTB: F:5′-AGATCAAGATCATTGCTCCTCCTG-3′, 24 bp; R:5′-TCACAGTCCGCCTAGAAGCA-3′, 20 bp. The relative expression level of the I*κ*B*α* gene mRNA was calculated and analyzed by the 2^−ΔΔCt^ method.

#### 2.4.3. Western Blot Analysis

Seventy-two hours after transfection, total protein was extracted from each group with RIPA lysates (Epizyme, China) containing protease inhibitors (Epizyme, China) (1 : 100) and nucleases (Haigene, China) (1 : 100) and quantified with BCA Protein Assay Kit (Cwbio, China). After each lane was loaded with 20 *μ*g protein by SDS-PAGE gel electrophoresis, the protein ladders were transferred to a PVDF membrane (Milipore, USA). And then, the membrane was blocked at room temperature for 5 minutes with a fast blocking solution (Epizyme, China) (1 : 100) and incubated with rabbit anti-monkey I*κ*B*α* monoclonal antibody (CST, USA) (1 : 5000) and rabbit anti-monkey *ß*-actin monoclonal antibody (CST, USA) (1 : 10000) overnight at 4°C. Then HRP-labeled goat anti-rabbit IgG secondary antibody (CST, USA) (1 : 5000) was added and incubated for 1 hour at room temperature. Specific signals were visualized with Immobilon Western chemiluminescence HRP Substrate (Milipore, USA) and acquired with ChemiDocTM Touch Imaging System (Bio-Rad, USA). The gray value of each band and the relative expression of I*κ*B*α* protein were calculated and analyzed by Gel-Pro analyzer software version 4.

### 2.5. Transfection Concentration Optimization

#### 2.5.1. Transfection Efficiency

Both cells were seeded in 6-well culture plates at about 5 × 10^6^ per well and were divided into 5 groups. The control group was transfected with 10 nM Cy5-NC-siRNA without transfection reagent, and the other four groups were transfected with different concentrations of Cy5-NC-siRNA (10, 20, 50, and 100 nM) combined with transfection reagent. Cy5-NC-siRNA was diluted to the above four concentrations with Opti-MEM, and the transfection was performed according to the above transfection steps. After 24 hours, the cells in the 6-well plate were digested into single-cell suspension and then were centrifuged at 1000 r/min for 5 minutes. The supernatant was discarded, and the cells were resuspended in phosphate-buffered saline (PBS) (Gibco, USA). The transfection efficiency was tested by flow cytometry (FACS Aria, BD, USA).

#### 2.5.2. Cytotoxicity

Both cells were seeded in 96-well culture plates at about 10^4^ per well and were divided into 5 groups. The control group was only transfected with transfection reagent, and the other four groups were transfected with different concentrations of I*κ*B*α*-siRNA (10, 20, 50, and 100 nM) combined with transfection reagent. The candidate valid I*κ*B*α*-siRNA was diluted of the above four concentrations with Opti-MEM, and transfection was performed according to the above transfection steps. 24 hours after transfection, 10 *μ*l of CCK8 (Dojindo, Japan) was added to each well. After 1 to 4 hours, the absorbance at 450 nm was measured with Synergy H1 Hybrid Microplate Reader (BioTek, USA), and the relative activities of cells in each group were calculated.

#### 2.5.3. Inhibitory Rate

Both cells were seeded in 6-well culture plates at about 5 × 10^6^ per well and were divided into 5 groups. The control group was only transfected with transfection reagent, and the other four groups were transfected with different concentrations of I*κ*B*α*-siRNA (10, 20, 50, and 100 nM) combined with transfection reagent. The candidate valid I*κ*B*α*-siRNA was diluted into the above four concentrations with Opti-MEM, and the transfection was performed according to the above transfection steps. After 24 hours, the total RNA of each group was extracted and reversely transcribed to generate cDNA. The relative expression of I*κ*B*α* mRNA was detected by using real-time PCR.

### 2.6. Statistical Analysis

Each individual in vitro experiment was tested independently and repeated 3 times. Statistical analyses were performed using Graph Pad Prism version 7.0. Multiple comparisons were made by one-way analysis of variance followed by Bonferroni-Dunn tests. The threshold for significance was set at *P* < 0.05.

## 3. Results

### 3.1. Culture and Identification of Monkey CM Cells and TM Cells

**Figure 1:**
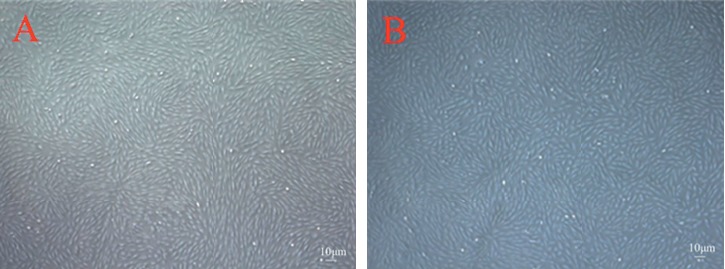
Subcultured cynomolgus monkey ciliary muscle (CM) cells and cynomolgus trabecular meshwork (TM) cells (×40). (a) The cultured CM cells were spindle-shaped and merged into a single layer to form a hill-and-valley pattern typical of human ciliary muscle cell in vitro. (b) The cultured TM cells are diverse in morphology between the epithelial cell type and the fibroblast type.

**Figure 2:**
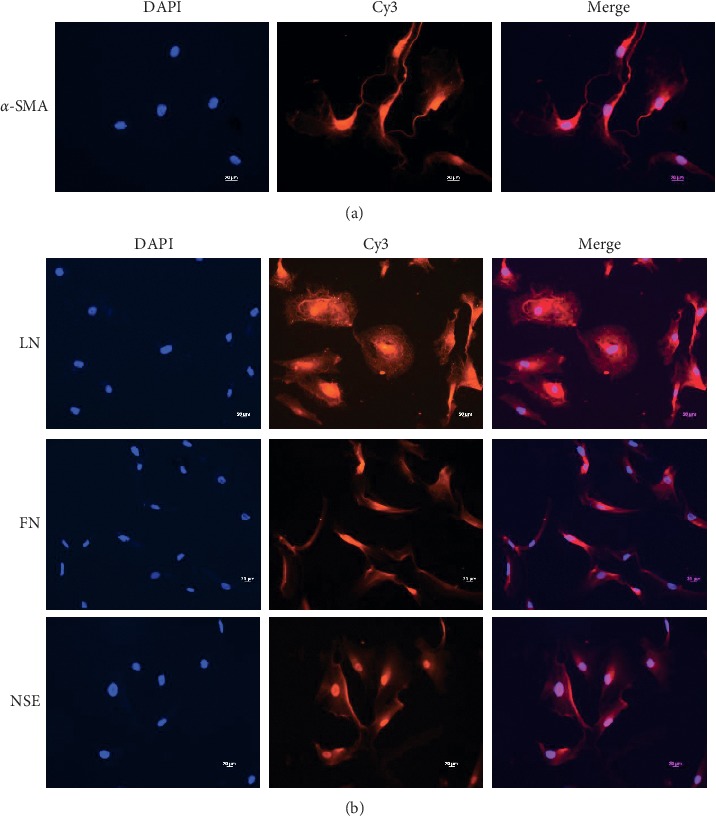
Identification of primary cultured cynomolgus monkey ciliary muscle cells and trabecular meshwork cells by immunofluorescence (×200). (a) The primary CM cells stained positively against *α*-SMA. (b) The primary TM cells stained positively against LN/FN/NSE.

The cultured CM cells were spindle-shaped and merged into a single layer to form a hill-and-valley pattern typical of human CM cell in vitro [12] (Figure 1(a)). Positive immunofluorescence staining of anti-*α*-SMA antibody showed that more than 95% of the cells were smooth-muscle cells, and negative control cultures showed no positive staining (Figure 2(a)). The cultured TM cells were diverse in morphology between the fibroblast type and the epithelial cell type [13](Figure 1(b)). Positive immunofluorescence staining of anti-LN/FN/NSE antibodies confirmed that more than 95% of the cells were TM cells, and negative control cultures showed no positive staining (Figure 2(b)).

### 3.2. In Vitro Screening of Effective I*κ*B*α*-siRNA Sequence of Cynomolgus Monkey

**Figure 3:**
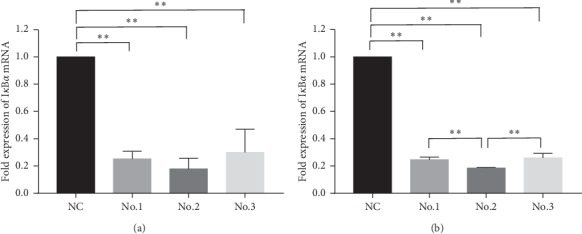
Real-time PCR for detection of I*κ*B*α* mRNA expression after NC-siRNA and three pairs of I*κ*B*α*-siRNA were transfected into CM cells and TM cells, respectively, at 10 nM. (a) The three pairs of I*κ*B*α*-siRNA could all inhibit the I*κ*B*α* gene expression, and the inhibitory rate of No.2 was higher than that of the other two pairs in CM cells. (b) The three pairs of I*κ*B*α*-siRNA could all inhibit the I*κ*B*α* gene expression, and the inhibitory rate of No.2 was higher than that of the other two pairs in TM cells. ^*∗*^*P* < 0.05; ^*∗∗*^*P* < 0.01.

**Figure 4:**
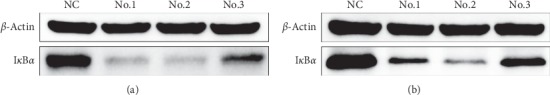
Western blotting for detection of I*κ*B*α* protein expression after NC-siRNA and three pairs of I*κ*B*α*-siRNA were transfected into CM cells and TM cells, respectively, at 10 nM. (a) The relative protein expression of I*κ*B*α* of No. 1, No. 2, and No. 3 groups were significantly lower compared to that of the NC-siRNA group in CM cells. The three pairs of I*κ*B*α*-siRNA could all inhibit the I*κ*B*α* protein expression, and the inhibitory rate of No. 2 was higher than that of the other two pairs. (b) The relative protein expression of I*κ*B*α* of No. 1, No. 2, and No. 3 group was significantly lower compared to that of the NC-siRNA group in TM cells. The three pairs of I*κ*B*α*-siRNA could all inhibit the I*κ*B*α* protein expression, and the inhibitory rate of No. 2 was higher than that of the other two pairs.

I*κ*B*α*/NC-siRNA was transfected into CM cells and TM cells at 10 nM. The expression of I*κ*B*α* mRNA and protein was detected by using real-time PCR and western blot at 48 hours and 72 hours, respectively, after transfection to seek for the siRNA sequences with the highest inhibitory rate targeting on cynomolgus I*κ*B*α* gene. The mRNA expression of I*κ*B*α* in each experimental group was 0.25 ± 0.06, 0.18 ± 0.08, and 0.30 ± 0.17 for CM cells (Figure 3(a)) and 0.25 ± 0.02, 0.18 ± 0.01, and 0.26 ± 0.03 for TM cells (Figure 3(b)). The protein expression of I*κ*B*α* in each experimental group was 0.04 ± 0.01, 0.02 ± 0.01, and 0.23 ± 0.17 for CM cells (Figure 4(a)) and 0.34 ± 0.15, 0.07 ± 0.05, 0.53 ± 0.08 for TM cells (Figure 4(b)). The results of both cells showed that at the mRNA and protein levels, the expression of I*κ*B*α* in each experimental group was significantly lower than that in the NC-siRNA group (*P* < 0.01), indicating that all of the three pairs of siRNA could inhibit the I*κ*B*α* gene and that the inhibitory rate of No. 2 was higher than that of the other two pairs, which reached 82% for CM cells and 82% for TM cells at the mRNA level and 98% for CM cells and 93% for TM cells at the protein level, respectively. Therefore, No. 2 I*κ*B*α*-siRNA was used in the follow-up experiments.

### 3.3. Transfection Concentration Optimization

#### 3.3.1. Transfection Efficiency

**Figure 5:**
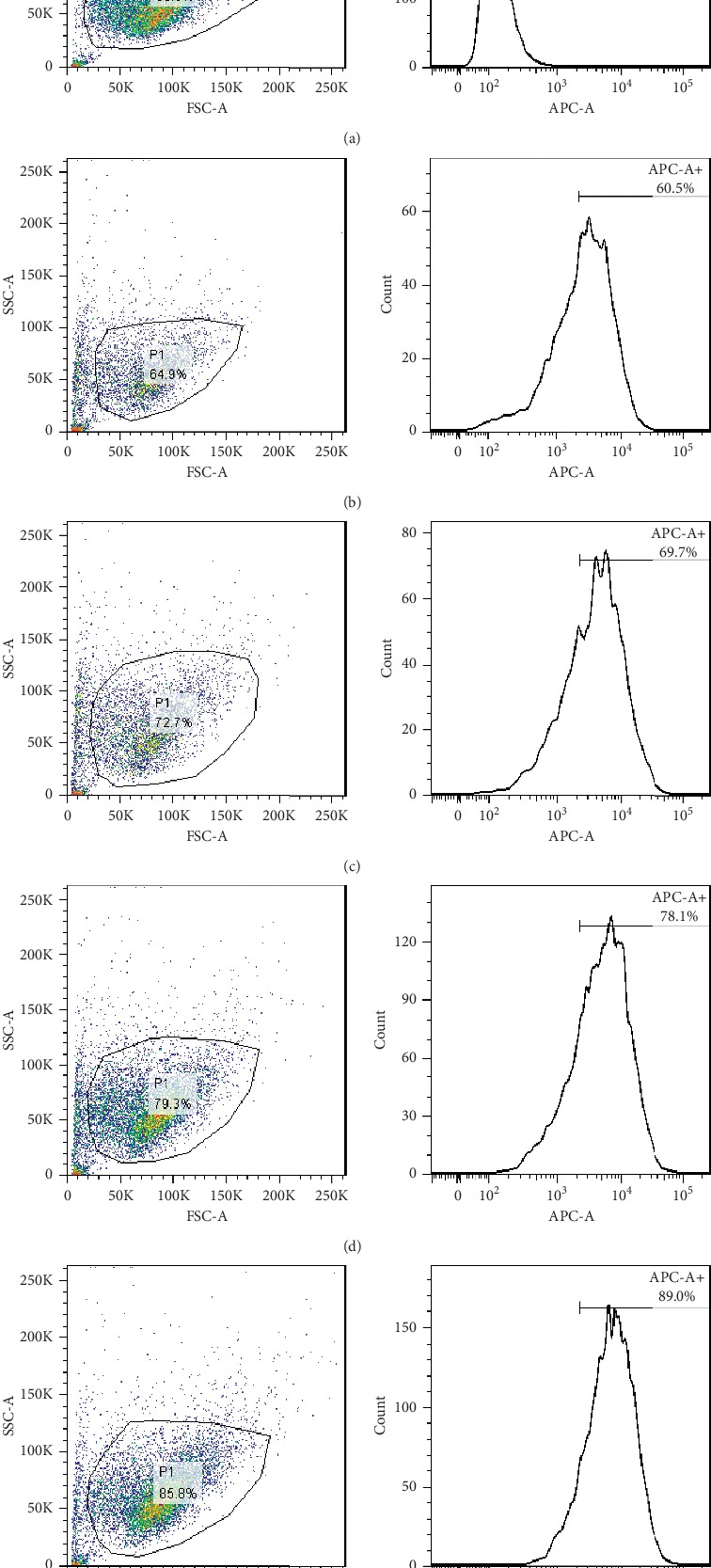
The transfection efficiency of different concentrations of siRNA in CM cells (APC-A+ indicates the number of cells labeled with Cy5). (a) Transfection of 10 nM Cy5-NC-siRNA control group. (b) Transfection reagent + 10 nM Cy5-NC-siRNA group. (c) Transfection reagent + 20 nM Cy5-NC-siRNA group. (d) Transfection reagent + 50 nM Cy5-NC-siRNA group. (e) Transfection reagent + 100 nM Cy5-NC-siRNA group. The higher the transfection concentration was, the more cells were labeled with Cy5, the higher the transfection efficiency was.

**Figure 6:**
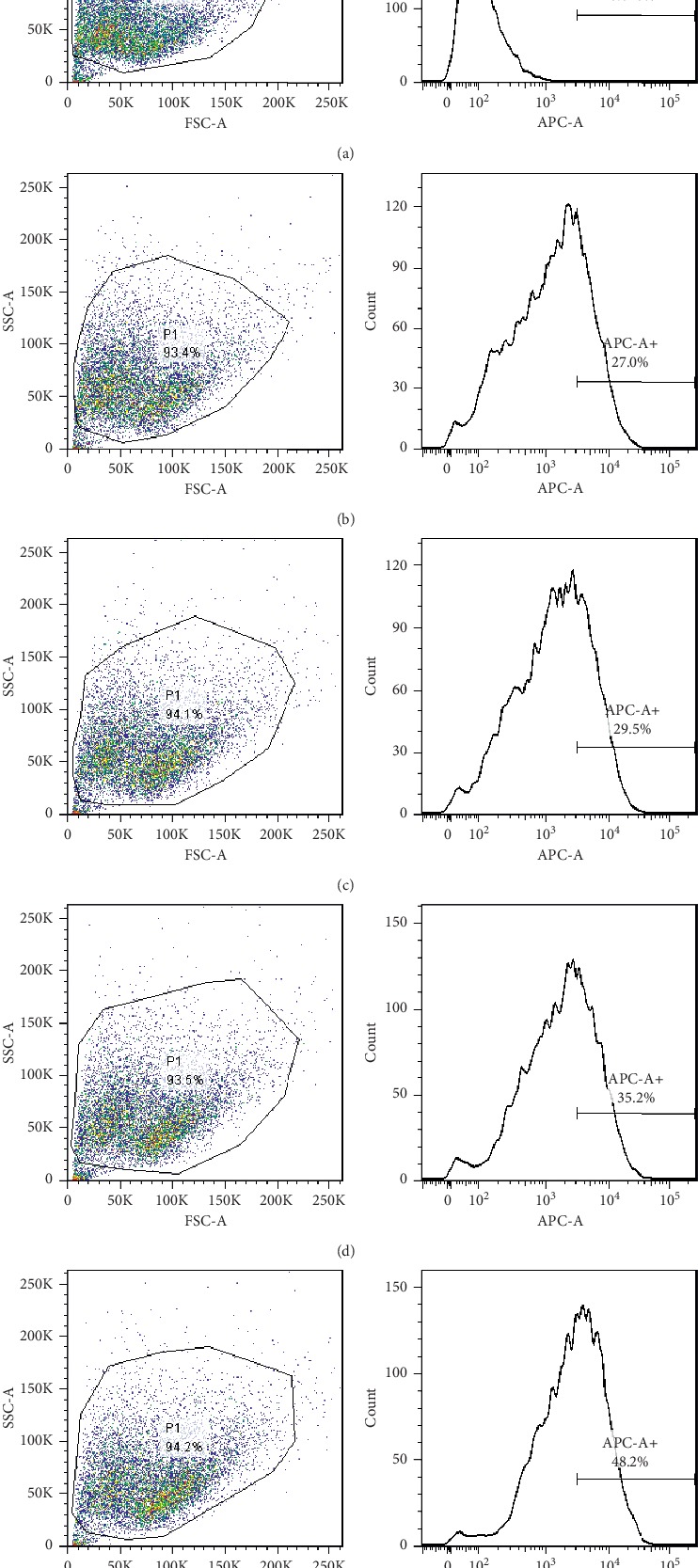
The transfection efficiency of different concentrations of siRNA in TM cells (APC-A+ indicates the number of cells labeled with Cy5). (a) Transfection of 10 nM Cy5-NC-siRNA control group. (b) Transfection reagent +10 nM Cy5-NC-siRNA group. (c) Transfection reagent + 20 nM Cy5-NC-siRNA group. (d) Transfection reagent + 50 nM Cy5-NC-siRNA group. (e) Transfection reagent + 100 nM Cy5-NC-siRNA group. The higher the transfection concentration was, the more cells were labeled with Cy5, the higher the transfection efficiency was.

Cy5-labeled NC-siRNA was transfected into CM cells and TM cells at the final concentration of 10, 20, 50, and 100 nM, respectively. After 24 hours, the transfection efficiency was detected by flow cytometry, which was 0% for the control group (Figure 5(a)), 60.5% for 10 nM group (Figure 5(b)), 69.7% for 20 nM group (Figure 5(c)), 78.1% for 50 nM group (Figure 5(d)), and 89.0% for 100 nM group (Figure 5(e)) for CM cells (Figure 5), and 0% for control group (Figure 6(a)), 27.0% for 10 nM group (Figure 6(b)), 29.5% for 20 nM group (Figure 6(c)), 35.2% for 50 nM group (Figure 6(d)), and 48.2% for 100 nM group (Figure 6(e)) for TM cells (Figure 6). It showed that the higher the transfection concentration was, the more cells were labeled with Cy5, the higher the transfection efficiency was.

#### 3.3.2. Cytotoxicity

**Figure 7:**
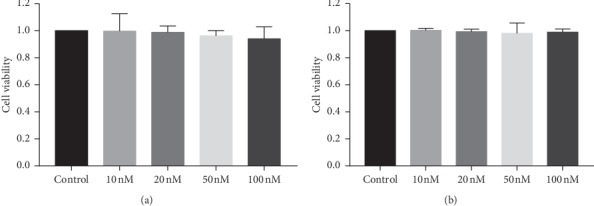
Relative activity of the cells after transfection of different concentrations of I*κ*B*α*-siRNA, ranged from 10 nM to 100 nM. The control group was only transfected with transfection reagent. (a) There was no statistically significant difference in each group for CM cells. (b) There was no statistically significant difference in each group for TM cells.

I*κ*B*α*-siRNA with the final concentration of 10, 20, 50, and 100 nM was transfected into CM cells and TM cells, respectively. Twenty-four hours later, after adding CCK8, the absorbance was measured by the microplate reader, and the relative activities of each group of cells were calculated, which were 1.00 ± 0.13, 0.99 ± 0.05, 0.96 ± 0.04, and 0.94 ± 0.09 for CM cells (Figure 7(a)) and 1.00 ± 0.01, 0.99 ± 0.08, 0.98 ± 0.08, and 0.99 ± 0.02 for TM cells (Figure 7(b)). The statistical results showed that there was no statistically significant difference in each group in both cells.

#### 3.3.3. Inhibitory Rate

**Figure 8:**
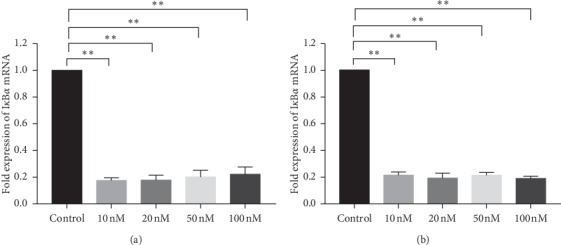
Relative expression of I*κ*B*α* mRNA after transfection of different concentrations of I*κ*B*α*-siRNA ranged from 10 nM to 100 nM. The control group was only transfected with transfection reagent. (a) The mRNA expression of I*κ*B*α* in each experimental group was significantly lower than that in the reagent transfection group (*P* < 0.01). However, no statistically significant associations were observed among different concentration groups ranged from 10 nM to 100 nM in CM cells. (b) The mRNA expression of I*κ*B*α* in each experimental group was significantly lower than that in the reagent transfection group (*P* < 0.01). However, no statistically significant associations were observed among different concentration groups ranged from 10 nM to 100 nM in TM cells. ^*∗*^*P* < 0.05; ^*∗∗*^*p* < 0.01.

No. 2 I*κ*B*α*-siRNA was transfected into CM cells and TM cells at the final concentration of 10, 20, 50, and 100 nM. After 24 hours, the expression of I*κ*B*α* mRNA was tested by using real-time PCR, which were 0.17 ± 0.02, 0.17 ± 0.04, 0.20 ± 0.05, and 0.22 ± 0.06 for CM cells (Figure 8(a)), and 0.21 ± 0.03, 0.19 ± 0.04, 0.21 ± 0.03, and 0.18 ± 0.02 for TM cells (Figure 8(b)). The results of both cells showed that the mRNA expression of I*κ*B*α* in each experimental group was significantly lower than that in the reagent transfection group (*P* < 0.01). However, no statistically significant associations were observed among different concentration groups, ranged from 10 nM to 100 nM in both cells, indicating that there was no statistical difference in the inhibitory rate of I*κ*B*α* after RNAi at different concentrations of I*κ*B*α*-siRNA, ranged from 10 nM to 100 nM.

## 4. Discussion

In this study, we successfully cultured the primary cynomolgus monkey CM cells and TM cells. It took approximately four to seven days for the primary CM cells to form a monolayer of spindle-shaped cells with hill-and-valley pattern typical of human CM cells in vitro as described by other investigators [12, 14–16]. To demonstrate that the cultured CM cells were not contaminated with fibroblasts, we used immunofluorescence staining with anti-*α*-SMA specific antibodies. The primary CM cells stained positively against *α*-SMA, and negative control cultures showed no positive staining, demonstrating that the cells we isolated were pure monkey CM cells. Meanwhile, the primary TM cells were broad, flat, and partially elongated, which coincided with the characteristics as previously reported [13, 17–20]. Trabecular meshwork cells have the properties of secreting FN and LN [21, 22]. Additionally, NSE is an enzyme expressed by neural tissue and the embryogenesis of trabecular meshwork is derived from neural crest. Therefore, expression of NSE is a characteristic of trabecular meshwork cells [23]. So, to demonstrate that the cultured TM cells we isolated were pure monkey TM cells, we used immunofluorescence staining with anti-LN/FN/NSE antibodies. The primary TM cells stained positively against LN/FN/NSE, and negative control cultures showed no positive staining, indicating that we have cultured the pure monkey TM cells.

RNAi, a sequence-specific posttranscriptional gene silencing, is a process in which a double-stranded RNA molecule shuts down or silences the expression of a corresponding sequence gene at the mRNA level. SiRNA of 21–23 nucleotides is the important effector molecule [24]. Because of its high efficiency, specificity, stable action, and simple operation, RNAi has been widely used in researching the molecular mechanism of intracellular signaling pathways. RNAi technology includes design and synthesis of siRNA, validation of in vitro assays, and in vivo experiments [25]. In this study, 3 pairs of siRNA against cynomolgus monkey I*κ*B*α* gene were synthesized and transfected into primarily cultured cynomolgus monkey CM cells and TM cells. The interference effects of I*κ*B*α* mRNA and protein levels were detected by using real-time PCR and western blot. The results showed that three pairs of siRNA all inhibited the expression of I*κ*B*α* mRNA and protein, and No. 2 I*κ*B*α*-siRNA targeting the GCACTTAGCCTCTATCCAT had the most obvious inhibitory effect.

Because siRNA is easily degradable [26, 27], Lipofectamine® RNAiMAX Reagent was used as the transfection reagent. Lipofectamine RNAiMAX Transfection Reagent, as an advanced and highly efficient siRNA delivery solution, provides superior gene knockdown with less siRNA. It has a simple protocol that eliminates the need for removing the transfection complex or change of media after transfection. It maintains excellent cell viability over a 10-fold reagent concentration range, allowing reagents to be easily optimized for the lowest siRNA concentration while reducing the cytotoxicity of the experimental system.

In this study, the transfection efficiency detected by flow cytometry was the highest at 100 nM. But we also tested the cellular activity through CCK8 and inhibitory efficiency of I*κ*B*α* via real-time PCR after transfection of different concentrations of I*κ*B*α*-siRNA. Our results have demonstrated that there was no statistically significant change of cellular activity in each group, suggesting that no matter what transfection concentration was used, the cell toxicity would not get influenced. We also found that there was no significant difference in the inhibitory efficiency of I*κ*B*α* among different concentration groups, ranged from 10 nM to 100 nM, meaning that RNAi could effectively inhibit the I*κ*B*α* mRNA and protein expression even at 10 nM. On the basis of those results, we can draw a conclusion that 10 nM is the optimal transfecting concentration in vitro, so the subsequent experiments can be continually performed with Lipofectamine RNAiMAX Transfection Reagent at a transfection concentration of 10 nM I*κ*B*α*-siRNA.

We still lack the results of the expression changes of NF-*κ*B, MMPs, and TIMPs in vitro after inhibition of I*κ*B*α*. We would continue to explore the changes of NF-*κ*B, MMPs, and TIMPs in monkey TM cells and CM cells after RNAi targeting I*κ*B*α*.

## 5. Conclusion

In conclusion, this experiment successfully screened and identified a pair of siRNA sequences, which can effectively inhibit the I*κ*B*α* gene of cynomolgus monkey CM cells and TM cells and the optimal transfection concentration in vitro. This study lays the foundation for subsequent in vitro experiments and regulation of I*κ*B*α*-siRNA in the aqueous humor outflow dual channels of cynomolgus monkeys in vivo experiments.

## Figures and Tables

**Table 1:** Cynomolgus Monkey IκBα gene siRNA sequences.

I*κ*B*α*-siRNA number	Target sequence	siRNA sequence (5′-3′)
No. 1	CCGAGACTTTCGAGGAAAT	CCGAGACUUUCGAGGAAAU dTdT
		AUUUCCUCGAAAGUCUCGG dTdT
No. 2	GCACTTAGCCTCTATCCAT	GCACUUAGCCUCUAUCCAU dTdT
		AUGGAUAGAGGCUAAGUGC dTdT
No. 3	GCTGATGTCAACAGAGTTA	GCUGAUGUCAACAGAGUUA dTdT
		UAACUCUGUUGACAUCAGC dTdT

## Data Availability

The data used to support the findings of this study are available within this article.

## Supplementary Materials

Supplementary Materials Supplementary Figure 1: negative controls of the staining used in the study of identification of cultured cells stained by PBS and secondary antibodies without primary antibody. (A) The CM cells showed no staining for PBS. (B) The TM cells showed no staining for PBS.

## References

[1] Mantravadi A. V., Vadhar N. (2015). Glaucoma. *Primary Care: Clinics in Office Practice*.

[2] Sharts-Hopko N. C., Glynn-Milley C. (2009). Primary open-angle glaucoma. *AJN, American Journal of Nursing*.

[3] Luna C., Li G., Liton P. B., Epstein D. L., Gonzalez P. (2009). Alterations in gene expression induced by cyclic mechanical stress in trabecular meshwork cells. *Molecular Vision*.

[4] Porter K. M., Epstein D. L., Liton P. B. (2012). Up-regulated expression of extracellular matrix remodeling genes in phagocytically challenged trabecular meshwork cells. *PLoS One*.

[5] De Groef L., Van Hove I., Dekeyster E., Stalmans I., Moons L. (2013). MMPs in the trabecular meshwork: promising targets for future glaucoma therapies?. *Investigative Opthalmology & Visual Science*.

[6] Nga A. D., Yap S. L., Samsudin A., Abdul-Rahman P. S., Hashim O. H., Mimiwati Z. (2014). Matrix metalloproteinases and tissue inhibitors of metalloproteinases in the aqueous humour of patients with primary angle closure glaucoma—a quantitative study. *BMC Ophthalmology*.

[7] Sharif N. A., Katoli P., Scott D. (2014). FR-190997, a nonpeptide bradykinin B2-receptor partial agonist, is a potent and efficacious intraocular pressure lowering agent in ocular hypertensive cynomolgus monkeys. *Drug Development Research*.

[8] Piotrowska A., Izykowska I., Podhorska-Okołów M., Zabel M., Dziegiel P. (2008). The structure of NF-*κ*B family proteins and their role in apoptosis. *Postȩpy Higieny I Medycyny Doświadczalnej*.

[9] Hayden M. S., Ghosh S. (2008). Shared principles in NF-κB signaling. *Cell*.

[10] Lan Y. Q., Zhang C., Xiao J. H. (2009). Suppression of I*κ*B*α* increases the expression of matrix metalloproteinase-2 in human ciliary muscle cells. *Molecular Vision*.

[11] Kashiwagi K., Jin M., Suzuki M., Tanaka Y., Iizuka Y., Tsukahara S. (2001). Isopropyl unoprostone increases the activities of matrix metalloproteinases in cultured monkey ciliary muscle cells. *Journal of Glaucoma*.

[12] Weinreb R. N., Kashiwagi K., Kashiwagi F., Tsukahara S., Lindsey J. D. (1997). Prostaglandins increase matrix metalloproteinase release from human ciliary smooth muscle cells. *Investigative Ophthalmology and Visual Science*.

[13] Clark A. F., Lane D., Wilson K., Miggans S. T., McCartney M. D. (1996). Inhibition of dexamethasone-induced cytoskeletal changes in cultured human trabecular meshwork cells by tetrahydrocortisol. *Investigative Ophthalmology &amp; Visual Science*.

[14] Lindsey J. D., Kashiwagi K., Kashiwagi F., Weinreb R. N. (1997). Prostaglandins alter extracellular matrix adjacent to human ciliary muscle cells in vitro. *Investigative Ophthalmology and Visual Science*.

[15] Lindsey J. D., Kashiwagi K., Kashiwagi F., Weinreb R. N. (1997). Prostaglandin action on ciliary smooth muscle extracellular matrix metabolism: implications for uveoscleral outflow. *Survey of Ophthalmology*.

[16] Kashiwagi K., Lindsey J. D., Kashiwagi F., Tsukahara S., Weinreb R. N. (1997). Calponin distribution in human ciliary muscle and other anterior segment tissues. *Investigative Ophthalmology &amp; Visual Science*.

[17] Weinreb R. N., Polansky J. R., Alvarado J. A., Mitchell M. D. (1988). Arachidonic acid metabolism in human trabecular meshwork cells. *Investigative Ophthalmology and Visual Science*.

[18] Steely H. T., Browder S. L., Julian M. B., Miggans S. T., Wilson K. L., Clark A. F. (1992). The effects of dexamethasone on fibronectin expression in cultured human trabecular meshwork cells. *Investigative Ophthalmology and Visual Science*.

[19] Yue B. Y. J. T., Kurosawa A., Elvart J. L., Elner V. M., Tso M. O. M. (1988). Monkey trabecular meshwork cells in culture: growth, morphologic, and biochemical characteristics. *Graefe’s Archive for Clinical and Experimental Ophthalmology*.

[20] Tamm E. R., Russell P., Johnson D. H., Piatigorsky J. (1996). Human and monkey trabecular meshwork accumulate alpha B-crystallin in response to heat shock and oxidative stress. *Investigative Ophthalmology &amp; Visual Science*.

[21] Tripathi B. J., Tripathi R. C., Chen J., Gotsis S., Li J. (2004). Trabecular cell expression of fibronectin and MMP-3 is modulated by aqueous humor growth factors. *Experimental Eye Research*.

[22] Hernandez M. R., Weinstein B. I., Schwartz J., Ritch R., Gordon G. G., Southren A. L. (1987). Human trabecular meshwork cells in culture: morphology and extracellular matrix components. *Investigative Ophthalmology and Visual Science*.

[23] Le Lièvre C. S., Le Douarin N. M. (1975). Mesenchymal derivatives of the neural crest: analysis of chimaeric quail and chick embryos. *Journal of Embryology and Experimental Morphology*.

[24] Zamore P. D., Tuschl T., Sharp P. A., Bartel D. P. (2000). RNAi: double-stranded RNA directs the ATP-dependent cleavage of mRNA at 21 to 23 nucleotide intervals. *Cell*.

[25] Alagia A., Eritja R. (2016). siRNA and RNAi optimization. *Wiley Interdisciplinary Reviews: RNA*.

[26] Kanasty R., Dorkin J. R., Vegas A., Anderson D. (2013). Delivery materials for siRNA therapeutics. *Nature Materials*.

[27] Kesharwani P., Gajbhiye V., Jain N. K. (2012). A review of nanocarriers for the delivery of small interfering RNA. *Biomaterials*.

