# Supplementary material for: In Vitro Screening and Transfection Concentration Optimization of Cynomolgus Monkey IκBα-siRNA
Source: J Ophthalmol. 2020 Apr 15;2020:1848540. doi: 10.1155/2020/1848540 (PMC7180988; doi:10.1155/2020/1848540)
Supplement: Supplementary Materials — Supplementary Figure 1: negative controls of the staining used in the study of identification of cultured cells stained by PBS and secondary antibodies without primary antibody. (A) The CM cells showed no staining for PBS. (B) The TM cells showed no staining for PBS. [file 1848540.f1.pdf]

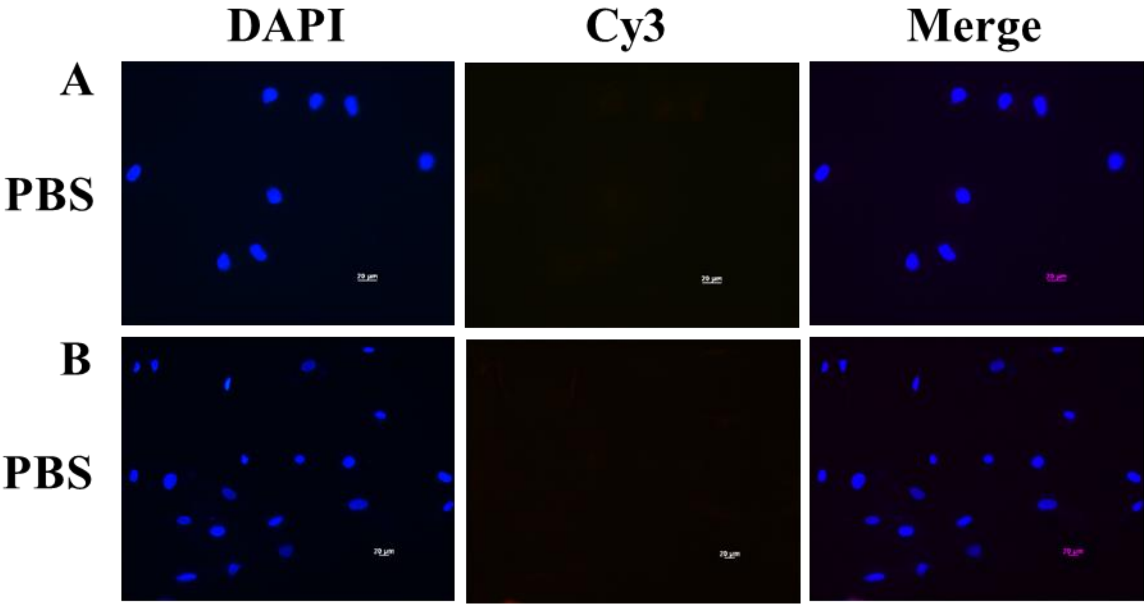

Supplementary Figure 1: negative controls of the staining used in the study of identification of cultured cells stained by PBS and secondary antibodies without primary antibody.

A. The CM cells showed no staining for PBS.

B. The TM cells showed no staining for PBS.
